# The Influence of Vegetation Height Heterogeneity on Forest and Woodland Bird Species Richness across the United States

**DOI:** 10.1371/journal.pone.0103236

**Published:** 2014-08-07

**Authors:** Qiongyu Huang, Anu Swatantran, Ralph Dubayah, Scott J. Goetz

**Affiliations:** 1 Department of Geographical Sciences, University of Maryland, College Park, Maryland, United States of America; 2 Woods Hole Research Center, Falmouth, Massachusetts, United States of America; University of Brasilia, Brazil

## Abstract

Avian diversity is under increasing pressures. It is thus critical to understand the ecological variables that contribute to large scale spatial distribution of avian species diversity. Traditionally, studies have relied primarily on two-dimensional habitat structure to model broad scale species richness. Vegetation vertical structure is increasingly used at local scales. However, the spatial arrangement of vegetation height has never been taken into consideration. Our goal was to examine the efficacies of three-dimensional forest structure, particularly the spatial heterogeneity of vegetation height in improving avian richness models across forested ecoregions in the U.S. We developed novel habitat metrics to characterize the spatial arrangement of vegetation height using the National Biomass and Carbon Dataset for the year 2000 (NBCD). The height-structured metrics were compared with other habitat metrics for statistical association with richness of three forest breeding bird guilds across Breeding Bird Survey (BBS) routes: a broadly grouped woodland guild, and two forest breeding guilds with preferences for forest edge and for interior forest. Parametric and non-parametric models were built to examine the improvement of predictability. Height-structured metrics had the strongest associations with species richness, yielding improved predictive ability for the woodland guild richness models (r^2^ = ∼0.53 for the parametric models, 0.63 the non-parametric models) and the forest edge guild models (r^2^ = ∼0.34 for the parametric models, 0.47 the non-parametric models). All but one of the linear models incorporating height-structured metrics showed significantly higher adjusted-r^2^ values than their counterparts without additional metrics. The interior forest guild richness showed a consistent low association with height-structured metrics. Our results suggest that height heterogeneity, beyond canopy height alone, supplements habitat characterization and richness models of forest bird species. The metrics and models derived in this study demonstrate practical examples of utilizing three-dimensional vegetation data for improved characterization of spatial patterns in species richness.

## Introduction

Avian diversity has been under increasing pressure from anthropogenic disturbances such as habitat loss and fragmentation [1]. Successful conservation planning relies upon understanding how the distribution of avian richness responds to existing and potential changes in environmental conditions which influence their distributions. Discovering the drivers of large-scale spatial variation of species richness has been a central debate in ecology [2]–[5], and many hypotheses have been proposed to address this issue [6]–[11]. One major hypothesis suggests that habitat heterogeneity is a key factor because it leads to greater spatial variability of habitat physical conditions, and therefore permits greater niche specialization resulting in more species richness [12]–[15]. Particularly in North America, habitat heterogeneity theory predicted the richness of some faunas significantly better than the species-energy theory [7], [14], [15]. This latter theory also has widespread support, and hypothesizes that productive energy through food webs or species physiological constraints to ambient energy determines species richness [4], [6], [8], [16].

Traditionally large scale habitat heterogeneity has been quantified mostly as topographical variability [7], [14], [17] or two dimensional habitat characteristics derived from remote sensing products [18], [19]. Vertical habitat structure may also lead to niche generalization, and as such be an important element of habitat heterogeneity affecting biodiversity [20]. However, it has rarely been used to explain species richness at broad scales. The incorporation of vertical heterogeneity is especially important for avian richness models where vertical habitat structure at local scales has long been recognized as a critical factor influencing bird life history [21]–[23] and abundance [24], [25].

Until recently, there have been relatively few studies utilizing three-dimensional habitat information due to difficulties of acquiring measurements of vertical vegetation structure beyond the plot scale over extended geographical areas [20]. This has changed significantly since the emergence of active remote sensing systems such as Light Detection and Ranging (lidar) and Radio Detection and Ranging (radar) which provide capability to map the vertical dimension of vegetation at local to regional scales [20], [26]. There is an increasing number of studies using lidar and radar derived three-dimensional vegetation structure to model biodiversity, many of which have revealed significant association between vegetation vertical structure, habitat quality, species richness and abundance [27]–[32]. However, none of the existing habitat metrics sufficiently characterize the spatial arrangement of vegetation height (i.e. the heterogeneity of height), nor its potential for predicting avian richness distributions over large geographical extents.

Related advances have been made in the development of statistical fusion models that provide a means to effectively combine remotely sensed data from radar, lidar, optical remote sensing systems and forest inventory data, yielding wall-to-wall high resolution vegetation structure maps at the continental scale [33]–[37]. The production of these maps not only enables the creation of habitat metrics that capture rich vegetation height heterogeneity, but also the comparison of the predictive abilities in various forms of these metrics. Our study is designed to embrace these opportunities by examining the relationship between forest bird richness, height-structured habitat metrics and avian richness models involving various degrees of forest height heterogeneity.

The overall goal of our study is to examine the potential of three-dimensional habitat structure in improving avian richness models at broad geographical scales. In doing so we hope to expand our understanding of the relationship between habitat structure and the spatial distribution of avian species richness, and to lay the foundation for constructing habitat metrics that better utilize increasingly available three-dimensional habitat data. Specifically we address the following questions:

1. How do the height-structured metrics compare with traditional habitat metrics in their ability to associate and predict forest bird richness in the forested ecoregion of the U.S.? Does incorporating the height-structured metrics improve the explanatory ability of avian richness models that use traditional habitat metrics?
2. How do the predictive abilities of richness models vary among forest bird guilds with contrasting preferences to habitat edges?

First, we introduce the conceptual similarities and differences between traditional habitat metrics and two types of height-structured metrics. Next, we describe the data and the methods we used to create the habitat metrics in this study. We then use correlation analysis and multivariate regression models to examine the relationships between different combinations of metrics and the species richness of three forest breeding bird guilds. Lastly, we examine the models’ explanatory abilities and the importance of individual metrics in predicting the richness of the three guilds.

## Background

**Figure 1:**
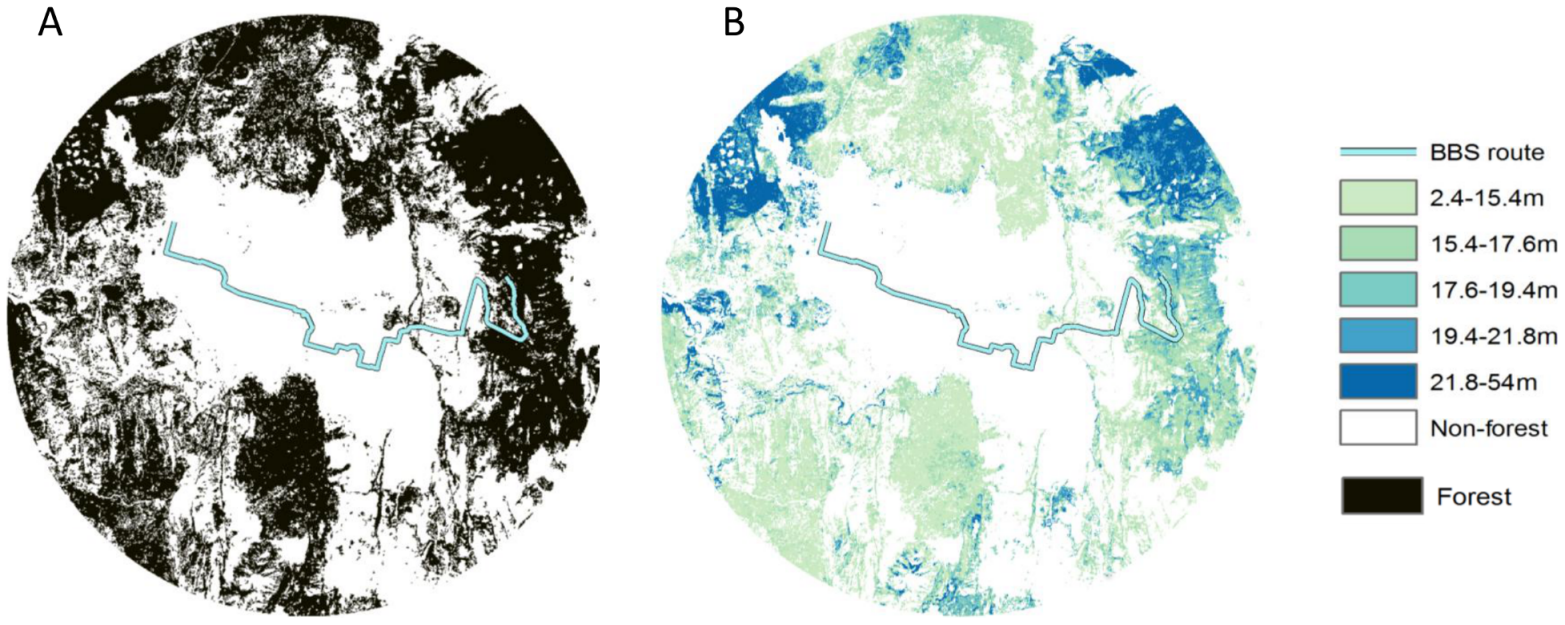
An example of the delineation of habitat patches at one BBS location. A two-dimensional vegetation map (A) and a vegetation map segmented by height structure (B) are shown. The pixel-based segmentation method (supporting information S1) is used to segment two dimensional habitat maps by using height thresholds.

Traditional habitat metrics are based primarily on two-dimensional habitat structure, such as land cover types, patch size and shape statistics. Developing such metrics generally depends on two steps: a) classifying scene space into binary habitat and non-habitat land cover types; b) delineating habitat patches based on the rule of contiguity (Figure 1) [38]. There have been numerous studies using habitat patch metrics and derivative habitat edge and contrast metrics to associate with ecological attributes such as species richness, reproductive success and individual fitness of birds [39]–[41]. However vegetation height information generally plays little role in the process of delineating habitat patches and characterizing their properties.

Some studies have applied three-dimensional habitat information in habitat quality and species diversity models [28], [30], [31], [42]–[44]. Usually, these applications rely on simple summary statistics such as mean, maximum, minimum and standard deviation to characterize three-dimensional vegetation structure. Summary statistics are straightforward and easy to obtain, but they cannot fully capture the heterogeneity of vegetation vertical structure. To give an example, one can have two forested landscapes with the same mean, maximum, minimum and standard deviation of tree height but with greatly different spatial arrangements of trees (e.g. tall trees can cluster in a few locations or can randomly distribute over the landscape which would have very different ecological implications for bird communities).

To account for more height heterogeneity, we created two groups of height-structured habitat metrics, the first of which integrates vegetation height information into the habitat patch framework while the second one characterizes canopy height distribution directly using second-order texture algorithms.

At the canopy level, vertical differences in vegetation create boundaries that segment contiguous habitats into smaller patches, each with similar height values (Figure 1). We first classified height pixels into a few height classes to characterize vertical edges and patches. Next, we grouped adjacent pixels from the same height class into patches. We treated the boundaries dividing those vertical patches as vertical edges (Figure 1). We also weighted the vertical edges by their depth (the height difference between two sides of a vertical edge) to capture the contrast of the height values of neighboring patches. By doing so, we could adapt a wide range of conventional habitat patch and edge metrics to account for complex spatial variability of canopy height.

Besides utilizing habitat patch and edge metrics to capture vegetation height heterogeneity, the second approach we introduce here involves calculation of the second-order (co-occurrence) texture statistics [45] directly from the gridded vegetation height maps. Second-order texture measures indicate the probabilities of each combination of pixel values co-occurring in a specific direction and distance [45]. These metrics can quantify spatial heterogeneity in terms of the spatial distribution and dependencies of height values [46] through grey level co-occurrence matrix. Texture measures are conventionally extracted from individual band of remotely sensed imagery and aerial photographs to assist object or land cover type discriminations [46], [47]. Normally a small moving window is used to calculate the grey level co-occurrence matrix in specified neighborhoods. Texture measures extracted from optical remote sensing imageries have been used to infer broadly defined habitat heterogeneity that includes various environmental factors (e.g. land cover type, vegetation type, soil condition as well as vertical structure). This type of habitat structural information has been linked to avian species richness in many studies [48]–[50]. Here we derived the second-order texture metrics from gridded canopy height maps to directly characterize habitat height structure and to associate them with variation in avian richness.

## Data Sets and Methods

### Avian Data

**Figure 2:**
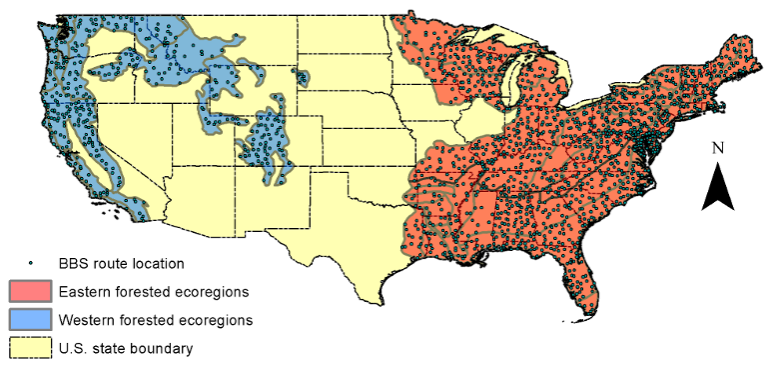
Distribution of BBS routes through the primarily forested ecoregions in the U.S. The richness models for the woodland guild were built using data from both eastern and western forested ecoregions. The forest edge and interior forest bird richness was modeled in the eastern forested ecoregions only.

The study area includes 21 predominately forested ecological regions (provinces) [51] across the conterminous U.S. (Table S1) (Figure 2). We used avian records from the Breeding Bird Survey (BBS) to model species richness over the entire study range. BBS is an annual road side survey organized by U.S. Geological Survey (USGS) [40], [52]. Initiated in 1966, BBS has over 4000 survey routes located on secondary roads across the continental U.S. and Canada. Each survey route is 39.4 km long. Every year, during the avian breeding season, surveys are conducted by competent volunteers using the protocol of three-minute point count at 50 stops at 0.8 km intervals. All birds seen or heard within 0.37 km radius are recorded [53]. We removed the records whose survey procedures or associated data are not acceptable by BBS standard. We also removed the records surveyed by first year observers to minimize observer bias [54]. We selected 134 broadly grouped woodland breeding birds species (here after “woodland guild”) based on the USGS species groupings [55]. We also selected 26 and 49 bird species as the forest breeding guilds with preference for interior forest habitat and forest edge habitat respectively (here after “interior forest guild” and “forest edge guild”) based on the classification of Boulinier et al.1998 [56]. A complete list of birds involved in this study and their guild assignment are given in Table S2. Because most of the interior forest and forest edge bird species are distributed in the Eastern U.S., we limited our analysis on these two guilds to the 10 forested ecoregions in the east (Figure 2).

Adjustments were made to take into account the detection probability bias [57]. We used the “fossil” package [58] in the R statistical program [59] to calculate the adjusted species richness using a first-order jackknife estimator [60], [61]. This estimator is based on multiple recapture studies in closed populations, which allows detection probability to vary among species. It is also the basic estimator underlying the species richness adjustments used by a USGS-developed BBS pre-processing program called COMDYN [62]. We averaged the available first-order jackknife richness within the five year period between 1998 and 2002 to temporally approximate the acquisition time of the radar data which played a key role in developing the vegetation height maps as discussed in the following section. The resulting mean avian richness is the richness we refer to in the rest of the study.

### Forest height data and habitat metrics

The National Biomass and Carbon Database of the year 2000 (NBCD) [33] provides an estimate of vegetation height distribution and variation at fine resolution for the conterminous U.S. The dataset is based on combined information from U.S. Department of Agriculture’s Forest Service Forest Inventory and Analysis data, high-resolution Interferometric Synthetic Aperture Radar data acquired from 2000 Shuttle Radar Topography Mission and optical remote sensing data from the Landsat ETM+ sensor. Products from the USGS’ National Land Cover Dataset 2001 and the Landscape Fire and Resource Management Planning Tools Project were also used during the process as input to build the empirical model for tree height estimation. The basal area weighted tree height (hereafter, “tree height”) maps produced by the model gives spatially explicit vegetation vertical structure maps over the conterminous U.S. of 30 m-resolution.

We adapted a method to use 19 km (∼half the length of a BBS route) radius buffers placed on the centroid of each BBS route, encompassing ∼1100 km^2^ areas to characterize the surrounding habitat around BBS locations [31], [63], [64]. We created habitat metrics on 1751 such circular landscapes where there are available BBS species richness data. (A), (B), (C), and (D), four metric sets incorporating a total of 26 metrics were calculated for each landscape (Table 1). The methods to produce each set of metrics are described in more details in the supporting information S1. The first two metric sets, embedded with little to no vegetation height heterogeneity, included (A) summary height statistics (hereafter “summary statistics”) and (B) traditional patch-based metrics. The other two metric sets incorporated height heterogeneity: (C) patch metrics characterizing vertical patches and edges (hereafter, “height-structured patch-based metrics”), and (D) second-order texture metrics capturing vertical heterogeneity of height distributions (Table 1). The metric sets (A) and (B) were created as baselines to compare with the height-structured metric sets (C) and (D).

**Table 1:** List of all metrics developed in the study.

Data	Metrics type (Set)	Metric name (Abbreviation)
NBCD vegetation height map	Summary height statistics (A)	Mean height (MEAN)
		Standard deviation of height (SD)
		Minimum height (Min)
		Maximum height (Max)
Two-dimensional vegetation cover map	Traditional patch-based metrics (B)	Number of patches(B.NP)
		Mean patch area (B.Area.MN)
		Standard deviation of patch area (B.Area.SD)
		Edge density (B. ED)
		Total edge (B.TE)
		Mean fractal dimension index (B.FRAC.MN)
		Standard deviation of fractal dimension index (B.FRAC.SD)
Vegetation cover map segmentedby height structure	Height-structured patch-based metrics (C)	Number of patches(C.NP)
		Mean patch area (C.Area.MN)
		Standard deviation of patch area (C.Area.SD)
		Total edge (C.TE)
		Mean fractal dimension index (C.FRAC.MN)
		Standard deviation of fractal dimension index (C.FRAC.SD)
		Contrast weighted edge density (C.CWED)
		Mean of edge contrast index(C.ECON.MN)
		Standard deviation of edge contrast index (C.ECON.SD)
		Shannon’s diversity index (C.SHDI)
NBCD vegetation height map	Second-order texture metrics (D)	Entropy
		Contrast
		Angular second moment (ASM)
		Homogeneity
		Dissimilarity

All the metrics created are listed in Table 1, and the detailed formula and descriptions for each metric are presented in Table S3. In order to differentiate the metrics with the same name from metrics set (B) and (C), capital letter “B” or “C” were given as prefixes to acronyms to indicate metric set membership (Table 1).

### Species Richness Models

We first explored the statistical correlation between richness of the three avian guilds and the habitat metrics to evaluate the association between individual habitat metrics and the richness of different guilds. The woodland species richness models were based on data of all 21 forested ecoregions, and the interior forest and forest edge guild models were limited to data of the 10 forested ecoregions from Eastern U.S. as noted earlier (Figure 2).

We selected 2 metrics from each of metric set (C) and (D) that on average had the best association with the richness of the three guilds as the best performing height-structured metrics (BPHMs). These four BPHMs were later combined with the traditional habitat metrics in multivariable models for comparisons of improvement. We limited our choice to only the four best metrics to avoid subsequent overfitting of our multivariate models while still maintaining enough representativeness.

We next constructed 6 multivariate linear models to explain each guild’s richness. The first 4 models were created using the complete list of metrics from set (A), (B), (C), and (D) respectively. They served to compare the explanatory abilities of models that characterize habitat condition with very different approaches. The two other models combined metric set (A) and (B) individually with the 4 BPHMs. We created the combined models to examine the impacts of adding spatial arrangement of height in richness models characterizing habitat in traditional ways.

We used a bootstrapping technique to provide the mean value and confidence intervals for the richness models’ adjusted-r^2^ values and AIC values to assess models’ explanatory ability and goodness of fit, as well as the variability of these measures. The bootstrap resampling was repeated 3000 times for each model. To examine the significance level of model improvements the 95% confidence interval of adjusted-r^2^ values and AIC values were obtained with the bias-corrected and accelerated (BCA) bootstrap algorithm [65] to make the interval’s median unbiased and adjusted for skewness.

Lastly we explored the effect of combining the 26 metrics from all four metric sets using a non-parametric Random Forest (RF) model. The RF model [66] is known for being able to handle large number of input variables without overfitting [67]. It is also well-suited for our study because the model allows for covariance between predictor variables, which commonly exists between different habitat metrics. The RF model also provides a mechanism for assessing predictor variable importance using a measure of cross-validated mean square error (out of bag mean square error (OOB MSE)). The higher the increase of OOB MSE (IncMSE) is, the more important a specific metric is. More detailed introduction of RF model is described in the supporting information S1. We also ran 6 RF models on the same combinations of metrics used by the linear models to compare the differences between linear and RF models. We set the number of trees to be 2000 for all models to allow for the mean residual error to converge. In our study the RF models were built with Random Forests package [68] in the R statistical program [59].

## Results

### Predictor metric correlation

**Figure 3:**
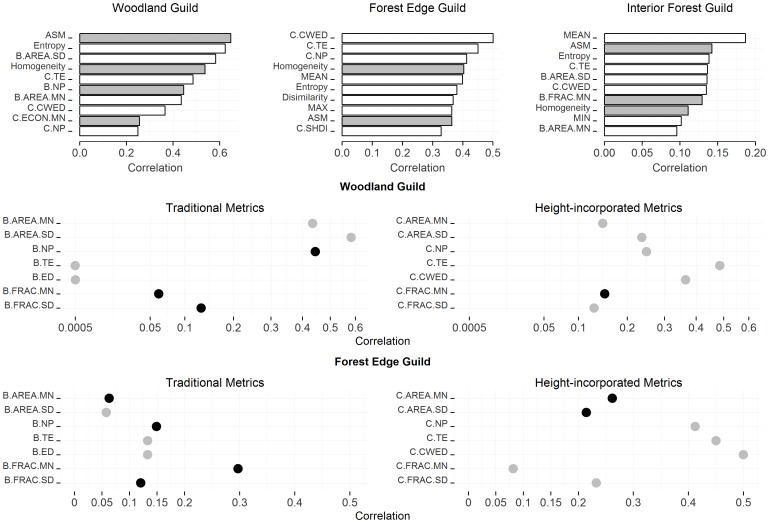
Guild richness associations with various metrics. (Top row): correlation bar plots of the most predictive metrics of species richness by guild. White bars represent a positive correlation and grey indicate a negative correlation. (Bottom rows): correlation comparisons between comparable patch-based metrics with and without considering the vertical patches and edges for the woodland and forest edge guild. The left panels show traditional metrics without accounting for height-heterogeneity; the right panels are height-structured counterparts. The black dots indicate a negative correlation and the grey ones indicate a positive correlation.

The predictor metrics that correlate best with bird species richness varied among guilds. For woodland species richness, (D) the second order texture metrics generally had the greatest predicative ability, followed by (B) the traditional patch-based metrics, and (C) the height-structured patch metrics. For forest edge bird richness, the metrics with the strongest correlation were (C) the height-structured patch-based metrics followed by (D) the second order texture metrics and (A) the summary height statistics (Figure 3). Interior forest bird species richness in general had consistently low correlation with any metrics. Among all the metrics developed (Table 1, Table S3), the metric with the greatest predictive capability for interior guild richness was mean vegetation height. ASM had the strongest average predictive capability over the richness models for three guilds, followed by entropy, C.TE, homogeneity, and C.CWED (Figure 3), all of which are height-structured metrics. We selected ASM, entropy, C.TE and C.CWED as the four BPHMs to be combined with models relied on the traditional metrics.

**Figure 4:**
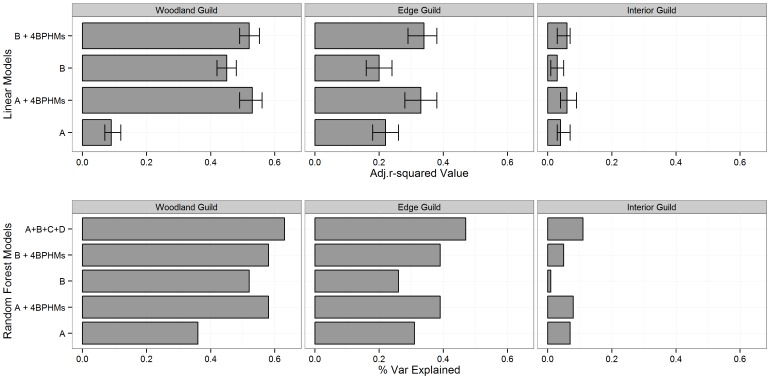
Predictive ability of multivariable models. A, B, C, and D are the four habitat metric sets, and 4BPHMs are the four best predictive height-structured metrics. Each of the top panels shows four linear models with whiskers giving 95% confidence interval of adjusted-r^2^ values. The length of the bar represents the mean adjusted-r^2^ for these models. The lower panels show the explained variance of the comparable random forest (RF) models. Uniquely the top bars at lower pannels are the results from the models employed all metrics from the four metric sets.

The direction of the correlation between metrics and the bird richness was generally consistent across three guild types except for metrics with weak correlation (Table S4). Among the variables with highest average correlation, ASM and homogeneity both had negative correlation with the richness of all three guilds. Conversely, entropy, C.TE, C.CWED all showed strong positive correlation for each guild’s richness (Figure 4, Table S4).

After incorporating vegetation height heterogeneity in patch-based metrics, the metrics characterizing patch number and area (AREA.MN, AREA.SD, and NP) showed a decreased correlation with the woodland guild richness. Conversely, the strength of the correlation between edge metrics (ED, TE) and the woodland guild richness increased. For the forest edge species both the patch and edge related metrics showed a prominent increase of correlation after incorporating vegetation height heterogeneity. The direction of the correlation for some patch-based metrics also changed. The NP metric showed an exceptionally large change for the woodland guild richness: from −0.45 to 0.25 after incorporating vertical patches (Table S4, Figure 3).

### Predictive models

**Figure 5:**
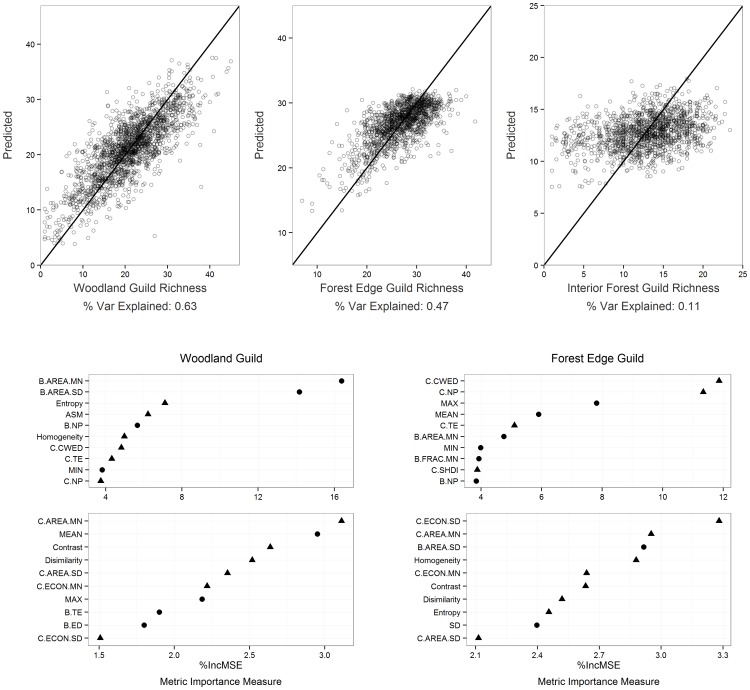
Random Forest model results. (Top row): Modeled vs. actual species richness for three guilds using all-inclusive random forest models. (Below the scatter plots): variable importance plots show the percent increase in mean square error (%IncMSE) of the top 20 most influential metrics in the woodland guild richness model and the forest edge guild richness model (note different scales on X-axes). The metrics characterizing vegetation height heterogeneity are plotted with triangles and the rest of the metrics are circles.

The non-parametric RF models combining all 26 metrics (all-inclusive models) from the four metric sets were the ones with greatest ability to predict species richness for each guild (Figure 4, Table S5). Among those models the lowest species richness variability was explained for the interior forest guild (r^2^ = 0.11), but the forest edge guild richness was predicted moderately well (r^2^ = 0.47) and the predictive model was strong for the woodland guild (r^2^ = 0.63) (Figure 5). The most important variable for predicting the woodland guild richness were two traditional patch-based metrics (B.AREA.MN and B.AREA.SD) followed by two second order texture metrics (entropy and ASM). The forest edge species richness model was most dependent on two height-structured patch metrics (C.CWED and C.NP) followed by two summary height statistics (MAX and MEAN). The most important predictive metrics for the interior forest guild model were MEAN followed by B.AREA.MN and B.AREA.SD (Figure 5).

For the RF models, our results consistently showed that adding height-structured metrics improved the model predictive ability. Specifically, the explained variance of the all-inclusive RF models for woodland and forest edge guild were up to 0.27 and 0.21 higher respectively than the RF models with only traditional habitat metrics. In addition for these two guilds, when the RF models were combined with the four BPHMs, the improvement for explained variance were up to 0.21 (woodland guild) and 0.13 (forest edge guild). The interior forest guild however showed only minor improvements when combined with any height-structured metrics. In general for woodland and forest edge guild, RF models’ predictabilities were higher than the comparable linear models by a prominent margin. (Figure 4 and Table S5).

The linear models had a lower explanatory ability than their RF counterparts. For a specific combination of habitat metrics, the linear models explained the most amount of variation in the woodland guild richness and the least in the interior forest guild richness. The one exception was the model using summary statistics of height (set A), which showed the highest predictability for forest edge guild richness, followed by woodland guild richness, and then the interior forest guild richness (Figure 4, Table S5). In every guild, the models incorporating the four BPHMs showed consistently higher predictability than the models without (Figure 4).

Combining the BPHMs with the summary height statistics resulted in significantly higher adjusted-r^2^ values in the woodland and forest edge models (Figure 4). The AIC value for the woodland richness model also improved significantly. In comparison, when combined with traditional patch-based metrics, the BPHMs significantly increased the adjusted-r^2^ for the forest edge guild model, while significantly improving the AIC values for both the forest edge and woodland guild models (Table S5).

## Discussion

A large number of hypotheses have been proposed to explain the spatial patterns of species richness over broad geographical scales [2], [4], [6], [69]–[71]. While it is unlikely that one single mechanism can explain species richness patterns completely, a large portion of the literature testing habitat heterogeneity hypothesis has focused on the association between species richness and two dimensional habitat structure, often combined with land cover type composition and distribution [63], [64], [72], [73]. On the other hand other studies testing species-energy hypothesis have relied on covariates related to ecosystem productivity and energy such as evapotranspiration and photosynthetic capacity indices like the normalized difference vegetation index (NDVI) [74]–[76] to explain large scale species richness patterns. Studies to associate habitat vertical structure with species richness are, however, often focused at local scale [31], which limited the efficacies of habitat heterogeneity models to explain species richness at broad scale.

Only recently was vegetation height information assessed as a predictor of avian species richness across the conterminous U.S. in two studies [31], [77]. One of these [31] used the same NBCD data we employed here, but they explored only summary statistics of vegetation height and biomass combined with land cover type composition and distribution. The other used sparsely sampled height metrics from a satellite lidar system that is no longer operating, and included climatic data as predictive variables [77]. Although our models employed only the distribution vegetation structures, with no input from other land cover type or climatic data, their explanatory ability for the woodland guild was comparable to these recent results (r^2^ = 0.70 for the forest guild model [31], and r^2^ = 0.60 for the open woodland model [77]). We found that models combining only vegetation vertical and horizontal structure can explain a significant amount of species richness for the broadly grouped woodland guild and the forest breeding guild with preferences for the forest edge habitat. More importantly, our results showed that incorporating vegetation vertical heterogeneity, and not just mean and standard deviation of height, greatly improves the ability to explain variability in avian richness for the two guilds. The spatial arrangement of vegetation height plays an important role in associating the quality of habitat condition and diversity of ecological niches for bird species within the two groups.

Traditionally habitat edges are thought to affect species movement, interaction, mortality and community dynamics [78]. The summary height statistics are considered indicators of habitat diversity and forest successional stage [20], [79], [80]. The traditional way of characterizing habitat through two-dimensional habitat patch distribution and summary height statistics still play important roles in our multivariate richness models. The large pool of traditional patch-based metrics provides a well-known framework to readily incorporate vertical height distribution once habitat patches are segmented by height. Both traditional and our height-structured metrics contribute to explanation of the variance of avian richness, although the importance of individual metrics in the models varies from guild to guild. Thus, our study shows that for the woodland avian guild and forest edge guild, the species richness is highly sensitive to the vegetation height heterogeneity, and the addition of the spatial arrangement of vegetation height provides significantly improved estimates of species richness for the two guilds. The patch-based height-structured metrics and the second order texture metrics thereby supplement and extend common methods of characterizing habitat condition and predicting avian species richness.

We note that the BBS data is collected along roadways where volunteers can easily and regularly traverse, thus the areas along the survey routes could be subject to disturbances such as motor vehicle traffic or habitat conversion [81], [82]; i.e. they may not be representative samplings of forest spatial and vertical variability. This characteristic of the data set could pose a challenge for systematical sampling of interior forest bird species in the surrounding areas and is likely one of the contributing factors for the consistently low species richness and weak correlations with our metrics and models in the case of the interior forest guild. Alternatively, forest edge habitats are relatively more exposed to stressors such as wind damages and human disturbances. They can exhibit higher vertical structure diversity than the interior forest areas [83]. It may be that interior birds are less adapted to habitat structure heterogeneity, and thus exhibit limited sensibility to habitat structure metrics. Lastly the results could also be attributed to the different ways members of avian guilds utilize habitat. Forest edge and majority of woodland bird species tend to use a wide range of habitat, and their degree of co-existence can vary in a broad spectrum over space. In comparison the interior forest guild, composed mostly of forest specialists that avoid other habitat types [84] with overlapping ecological niches, are more likely to face greater interspecific competition which limits species richness despite diverse height structure across landscapes [85]. However while the models see low association between height heterogeneity metrics and interior forest guild richness, there are still likely more specific vertical structure preferences associated with individual bird species [30], [86].

While four BPHMs highlighted in our study showed a good ability to associate with species richness and to improve broad scale avian richness modeling, it is reasonable to assume that height-structured metrics have potential to be improved further given the large number of options that remained unexplored. First, the pixel-based segmentation method used in our study (supporting information S1) is one of the simplest algorithms to delineate vertical patches and edges. The method is based on a set of global threshold values while not considering neighboring heterogeneity [87]. The process of setting up the threshold values and weight matrix (for contrast metrics) inevitably involves somewhat arbitrary decisions. More complex segmentation methods such as edge and region-based methods can be performed readily with commercial and open-source software packages that potentially may produce more efficacious vertical patches and be less arbitrary [88]. Secondly, there are many untested texture measures [45]. The relationship between texture metrics and the avian richness varies as the size of moving window changes [48]. More work is needed to understand the impact of those methodological options for further improving species richness models.

## Conclusions

As active remote sensing technologies like radar and lidar mature and become more widely available, data sets characterizing vegetation vertical structure should become increasingly useful for biodiversity applications and management. Our study showed that vegetation height heterogeneity is associated with habitat diversity and species richness for some forest avian guilds. Thus, while recognizing the advances conveyed by incorporating height information, there is an imperative to explore in more depth the role of such heterogeneity. Furthermore we suggest not just height, but vertical canopy heterogeneity, e.g. foliar profiles and layering, will provide an even richer source of information from which to develop new metrics and models [30], [83]. Incorporating such information will require data on not only canopy height but canopy vertical structure, the latter of which is unavailable at continental scales. Nonetheless, the metrics and models used in our analyses provide a means to incorporate and utilize three-dimensional habitat information, with the goal of better understanding the controls on avian species richness and habitat use.

## Supporting Information

Table S1 **Ecological provinces involved and the sub-region assignment.**
(XLSX)

Table S2 **Species list and guild classification.**
(XLSX)

Table S3 **Metric descriptions.**
(DOCX)

Table S4 **Metrics correlation with species richness.**
(XLSX)

Table S5 **Model performances for different multivariable models.**
(DOCX)

Supporting Information S1 **Contrast matrix for weighted-edge metrics.**
(PDF)
