# Supplementary material for: The Influence of Vegetation Height Heterogeneity on Forest and Woodland Bird Species Richness across the United States
Source: PLoS One. 2014 Aug 7;9(8):e103236. doi: 10.1371/journal.pone.0103236 (PMC4125162; doi:10.1371/journal.pone.0103236)
Supplement: Table S3 — Metric descriptions. (DOCX) [file pone.0103236.s003.docx]

| **Data used** | **Metrics type** | **Metrics name** | **Description** | **Notes** |
| --- | --- | --- | --- | --- |
| **NBCD vegetation height map** | Summary statistics (A) | Mean | $\frac{\sum x_{k}}{K}$ | $x_{k}$ = height values of forested pixels in the landscape. $K$ = the number of those pixels in the landscape. |
|  |  | Standard deviation (SD) | $\sqrt{\frac{{\sum(x_{k}-Mean)}^{2}}{K}}$ | N/A |
|  |  | Minimum (Min) | MIN{$X_{k}$} | N/A |
|  |  | Maximum (Max) | MAX{$X_{k}$} | N/A |
| **Two-dimensional vegetation cover map** | Traditional patch-based metrics (B) | Number of patches(B.NP) | Total number of patches in the landscape | N/A |
|  |  | Mean patch area (B.Area.MN) | $\frac{\sum x_{i}}{N}$ | $x_{i}$ = patch area across all patches in the landscape. $N$ = total number of patches. |
|  |  | Standard deviation of patch area (B.Area.SD) | $\sqrt{\frac{\sum{{(x}_{i}-Area.MN)}^{2}}{N}}$ | N/A |
|  |  | Edge density (B. ED) | $\frac{\sum_{k=1}^{m} e_{ik}}{A}(10,000)$ | $e_{ik}$ = total length of edge in landscape between height class i and k. A = total landscape area (m^2^). |
|  |  | Total edge (B.TE) | Total length (m) of edge in landscape | N/A |
|  |  | Mean fractal dimension index (B.FRAC.MN) | $\frac{\sum{2\ln(0.25p_{i})}/{lna_{i}}}{N}$ | $p_{i}$= perimeter of patch i. $a_{i}$ = area of patch i. |
|  |  | Standard deviation of fractal dimension index (B.FRAC.SD) | $\sqrt{\frac{\sum{{(f}_{i}-FRAC.MN)}^{2}}{N}}$ | $f_{i}$ = fractal dimension index across all patches. |
| **Vegetation cover** map **segmented by height structure** | Height-incorporated patch-based metrics (C) | Number of patches(C.NP) | Total number of patches in the landscape | patches and edges referred to for height-incorporated patch-based metrics (C) include vertical patches and edges. |
|  |  | Mean patch area (C.Area.MN) | $\frac{\sum x_{i}}{N}$ | $x_{i}$ = patch area across all patches in the landscape. $N$ = total number of patches. |
|  |  | Standard deviation of patch area (C.Area.SD) | $\sqrt{\frac{\sum{{(x}_{i}-Area.MN)}^{2}}{N}}$ | N/A |
|  |  | Edge density (C.B. ED) | $\frac{\sum_{k=1}^{m} e_{ik}}{A}(10,000)$ | $e_{ik}$ = total length of edge in landscape between height class i and k. A = total landscape area (m^2^). |
|  |  | Total edge (C.TE) | Total length (m) of edge in landscape | N/A |
|  |  | Mean fractal dimension index (C.FRAC.MN) | $\frac{\sum{2\ln(0.25p_{i})}/{lna_{i}}}{N}$ | $p_{i}$= perimeter of patch i. $a_{i}$ = area of patch i. |
|  |  | Standard deviation of fractal dimension index (C.FRAC.SD) | $\sqrt{\frac{\sum{{(f}_{i}-FRAC.MN)}^{2}}{N}}$ | $f_{i}$ = fractal dimension index across all patches. |
|  |  | Contrast weighted edge density (C.CWED) | $\frac{\sum\sum e_{ik}*d_{ik}}{A}(10,000)$ | $e_{ik}$ = total length of edge in landscape between height class i and k. $d_{ik}$= contrast weight between class i and k. A = total landscape area. |
|  |  | Mean of edge contrast index(C.ECON.MN) | $\frac{\sum\frac{\sum p_{ijk}*d_{ijk}}{P_{ij}}(100)}{N}$ | $p_{ijk}$=length of edge of patch ij adjacent to height class k. $d_{ijk}$= edge contrast weight between height class i and k. $P_{ij}$ =length of perimeter of patch ij. |
|  |  | Standard deviation of edge contrast index (C.ECON.SD) | $\sqrt{\frac{\sum{{(ECON}_{i}-ECON.MN)}^{2}}{N}}$ | ${ECON}_{i}$ =edge contrast index of patch i. |
|  |  | Shannon’s diversity index (C.SHDI) | $-\sum{Pr}_{i}*\ln{Pr}_{i}$ | ${Pr}_{i}$= proportion of the landscape occupied by height class i. |
| **NBCD vegetation height map** | Second-order texture measures (D) | Entropy | $-\sum_{i} \sum_{j} p\left( i,j \right)\log(p(i,j))$ | $p\left( i,j \right)$ is the $\left( i,j \right)$th entry of the normalized GLCM matrix. |
|  |  | Contrast | $\sum_{n=0}^{N-1} n^{2}\begin{matrix} \left\{ \sum_{i=1}^{N} \left. \sum_{j=1}^{N} p(i,j) \right\} \right. \\ \left\vert i-j \right\vert=n \end{matrix}$ | N/A |
|  |  | Angular second moment (ASM) | $\sum_{i} \sum_{j} \left. \left\{ p(i,j) \right. \right\}^{2}$ | N/A |
|  |  | Homogeneity | $\sum_{i} \sum_{j} \frac{p(i,j)}{1+\vert i-j\vert}$ | N/A |
|  |  | Dissimilarity | $\sum_{n=0}^{N-1} n \left\{ \sum_{i=1}^{N} \sum_{j=1}^{N} p(i,j) \right\}$ | N/A |
