# Supplementary material for: The Influence of Vegetation Height Heterogeneity on Forest and Woodland Bird Species Richness across the United States
Source: PLoS One. 2014 Aug 7;9(8):e103236. doi: 10.1371/journal.pone.0103236 (PMC4125162; doi:10.1371/journal.pone.0103236)
Supplement: Table S5 — Model performances for different multivariable models. (DOCX) [file pone.0103236.s005.docx]

Table S5. Model performances for different multivariable models.

| Model (number of variables) | Model Type | Measurement name | Woodland guild | Forest edge guild | Interior forest guild |
| --- | --- | --- | --- | --- | --- |
| D (5) | Linear | adj. r-squared | 0.48 | 0.26 | 0.06 |
|  |  | adj. r-squared 95% CI | 0.45 | 0.22 | 0.03 |
|  |  |  | 0.52 | 0.30 | 0.08 |
|  |  | AIC | 10783.64 | 7642.58 | 7721.88 |
|  |  | AIC 95% CI | 10663.48 | 7543.14 | 7626.97 |
|  |  |  | 10919.38 | 7772.84 | 7827.75 |
| C (10) | Linear | adj. r-squared | 0.45 | 0.30 | 0.04 |
|  |  | adj. r-squared 95% CI | 0.41 | 0.25 | 0.02 |
|  |  |  | 0.49 | 0.34 | 0.06 |
|  |  | AIC | 10886.77 | 7579.35 | 7745.47 |
|  |  | AIC 95% CI | 10776.59 | 7491.13 | 7655.00 |
|  |  |  | 11031.62 | 7708.06 | 7864.19 |
| A (4) | Linear | adj. r-squared | 0.09 | 0.22 | 0.04 |
|  |  | adj. r-squared 95% CI | 0.07 | 0.18 | 0.02 |
|  |  |  | 0.12 | 0.26 | 0.07 |
|  |  | AIC | 11768.01 | 7713.44 | 7737.10 |
|  |  | AIC 95% CI | 11650.73 | 7612.97 | 7645.61 |
|  |  |  | 11894.79 | 7832.26 | 7845.43 |
| B (7) | Linear | adj. r-squared | 0.45 | 0.20 | 0.03 |
|  |  | adj. r-squared 95% CI | 0.42 | 0.16 | 0.01 |
|  |  |  | 0.48 | 0.24 | 0.05 |
|  |  | AIC | 10894.73 | 7754.34 | 7757.22 |
|  |  | AIC 95% CI | 10779.40 | 7645.87 | 7661.07 |
|  |  |  | 11030.33 | 7881.45 | 7864.17 |
| A+4BPHM (8) | Linear | adj. r-squared | 0.52 | 0.33 | 0.06 |
|  |  | adj. r-squared 95% CI | 0.48 | 0.28 | 0.04 |
|  |  |  | 0.56 | 0.38 | 0.09 |
|  |  | AIC | 10642.09 | 7505.54 | 7713.40 |
|  |  | AIC 95% CI | 10515.22 | 7407.62 | 7621.75 |
|  |  |  | 10792.03 | 7632.99 | 7825.33 |
| B+4BPHM (11) | Linear | adj. r-squared | 0.52 | 0.34 | 0.06 |
|  |  | adj. r-squared 95% CI | 0.49 | 0.29 | 0.03 |
|  |  |  | 0.55 | 0.38 | 0.07 |
|  |  | AIC | 10648.74 | 7494.67 | 7727.33 |
|  |  | AIC 95% CI | 10544.73 | 7402.19 | 7636.25 |
|  |  |  | 10795.68 | 7622.01 | 7839.27 |
| A+B+C+D (26) | Random Forest | % variance explained | 0.63 | 0.47 | 0.11 |
| A (4) | Random Forest | % variance explained | 0.37 | 0.31 | 0.07 |
| A+4BPHM (8) | Random Forest | % variance explained | 0.58 | 0.39 | 0.08 |
| B (7) | Random Forest | % variance explained | 0.52 | 0.26 | 0.01 |
| B+4BPHM (11) | Random Forest | % variance explained | 0.58 | 0.39 | 0.05 |
| C (10) | Random Forest | % variance explained | 0.58 | 0.39 | 0.06 |
| D (5) | Random Forest | % variance explained | 0.50 | 0.23 | ~0.00 |
