# Supplementary material for: The Influence of Vegetation Height Heterogeneity on Forest and Woodland Bird Species Richness across the United States
Source: PLoS One. 2014 Aug 7;9(8):e103236. doi: 10.1371/journal.pone.0103236 (PMC4125162; doi:10.1371/journal.pone.0103236)
Supplement: Supporting Information S1 — Contrast matrix for weighted-edge metrics. (PDF) [file pone.0103236.s006.pdf]

Supporting Information S1:

Metrics development methods

Summary statistics

The summary statistics used in this study included, mean, standard deviation (SD), minimum value (Min), and maximum value (Max) of tree height (Table S3). All the metrics were computed in a GIS environment using ArcGIS 9.3 [1].

Patch-based metrics

Patch-based metrics based on two-dimensional vegetation distribution were derived by classifying the NBCD forest height maps into binary forest-non-forest maps. Vegetation height values larger than 0 were assigned as habitat and the rest of the cells as non-habitat, and vertical height information were discarded (Figure 2). Within each landscape a set of patch-based metrics (Table S3) were calculated using Fragstats [2]. The metrics in this group included patches (NP), mean patch area (Area.MN), standard deviation of patch area (Area.SD), mean fractal dimension index (FRAC.MN), standard deviation of fractal dimension (FRAC.SD), total edge (TE) and edge density (ED) where fractal dimension index was a measure of complexity of shape based on perimeter-area relationships.

Height-structured patch-based metrics

We used a pixel-based segmentation approach to create vertical patches and edges within continuous habitat patches. The pixel-based approach relies on a set of global thresholds to classify pixels into different groups, and then spatially connected pixels from the same group are segmented from the surrounding areas and assigned with different group memberships. The

classification process only depends on the attribute value of a specific pixel. It is a relatively simple method compared with edge-based and region-based methods which consider variation of attribute values in the neighboring regions when assigning group memberships [3]. We selected a set of thresholds using quintile classification based on frequency of height values across 1751 landscapes. The forest height values were classified into five groups using four thresholds: 20, 40, 60, and 80 percentile values at 15.4, 17.6, 19.4, and 21.8 m respectively (Figure 2).

A set of metrics similar to the ones in metric set (B) was then derived. A diversity index (Shannon's diversity index (SHDI)) was included to measure the richness of height classes, and a contrast weight matrix (see Table below) was used to calculate four adjacent contrast metrics. The contrast matrix is symmetrical with weights ranging from 0 (no contrast) to 1 (maximum contrast). When adjacent areas have great height differences, vertical edges separating the areas are weighted heavily and thus have a large contribution to the weighted edge measurements (Table S3). The resulting contrast weighted edge density (CWED) was derived from sum of weighted edges at the landscape level. Mean and standard deviation of an edge contrast index (ECON.MN & ECON.SD)) summarizes the distribution of weighted edge lengths of all patches, including vertical patches within a specific landscape [2].

Second-order texture metrics

We calculated a set of second-order texture metrics using the texture filter in ENVI 4.7 [4]. A variety of texture metrics have been used to characterize habitat structure [5]. We developed five texture measures: angular second moment (ASM), contrast, homogeneity, entropy, and dissimilarity. The metrics were developed by processing the NBCD tree height map with a 3 × 3

moving window. For our purposes, non-forest land cover types were reclassified as having 0 height value.

Random Forest Model

In essence, RF model is an ensemble machine learning technique which consists of a compilation of multiple regression trees, a method proven to be better than the single regression tree analysis [6]. The regression tree method seeks to construct a set of binary splits on the predictor variables; the dependent variable is predicted through recursive partitioning. The selected splits are the ones that maximize the homogeneity of the two resulting groups. In comparison, each tree in random forests method is built with a subset of variables randomly chosen. At each node, the number of predictors tested for the best split is also randomized. Like bagging trees method [7], the trees are grown to maximum size without pruning.

About 37% of data are randomly drawn as the out-of-bag (OOB) observations and excluded in the construction of every tree. The OOB error estimation provides a cross-validation mechanism, thus test sets or extra cross-validation is not necessary. The performance of the random forest models was estimated using percent of variance explained: R^2= 1- MSE/ observed variance, where MSE is mean square error between OOB predictions and observations[8,9].

References

1. Environmental Systems Research Institute (1999) Arc GIS. ESRI Redlands, California, USA.

2. McGarigal K, Cushman SA, Neel MC, Ene E (2002) FRAGSTATS: Spatial Pattern Analysis Program for Categorical Maps. Amherst: University of Massachusetts. Available: http://www.umass.edu/landeco/research/fragstats/fragstats.html.

3. Darwish A, Leukert K, Reinhardt W (2003) Image segmentation for the purpose of object-based classification. Geoscience and Remote Sensing Symposium, 2003. IGARSS '03. Proceedings. 2003 IEEE International. Vol. 3. pp. 2039–2041. doi:10.1109/IGARSS.2003.1294332.

4. Exelis Visual Information Solutions (Boulder, Colorado) ENVI. Exelis Visual Information Solutions.

5. St-Louis V, Pidgeon AM, Radeloff VC, Hawbaker TJ, Clayton MK (2006) High-resolution image texture as a predictor of bird species richness. Remote Sens Environ 105: 299–312. doi:10.1016/j.rse.2006.07.003.

6. Hamza M, Larocque D (2005) An empirical comparison of ensemble methods based on classification trees. J Stat Comput Simul 75: 629–643. doi:10.1080/00949650410001729472.

7. Breiman L (1996) Bagging predictors. Mach Learn 24: 123–140. doi:10.1007/BF00058655.

8. Breiman L (2001) Random Forests. Mach Learn 45: 5–23.

9. Wei C-L, Rowe GT, Escobar-Briones E, Boetius A, Soltwedel T, et al. (2010) Global Patterns and Predictions of Seafloor Biomass Using Random Forests. PLoS ONE 5: e15323. doi:10.1371/journal.pone.0015323.

Table. Contrast matrix for weighted edge metrics

Forest Height Class	NO.1 (2.8-15.4m)	NO.2 (15.4-17.6m)	NO.3 (17.6-19.4m)	NO.4 (19.4-21.8m)	NO.5 (21.8-53.7m)	Non-forest
NO.1	0	0.25	0.5	0.75	1	0.25
NO.2	-	0	0.25	0.5	0.75	0.5
NO.3	-	-	0	0.25	0.5	0.75
NO.4	-	-	-	0	0.25	1
NO.5	-		-	-	0	1
Non-forest	-	-	-	-	-	0
